# Assessing response, remission and treatment resistance in patients with Obsessive-Compulsive Disorder with and without Tic Disorders: results from a multicenter study

**DOI:** 10.1192/j.eurpsy.2022.1648

**Published:** 2022-09-01

**Authors:** D. Conti, N. Girone, B. Benatti, O. Gambini, U. Albert, G. Maina, M. Amore, M. Porta, B. Dell’Osso

**Affiliations:** 1 University of Milan, Luigi Sacco University Hospital, Psychiatry 2 Unit, Milan, Italy; 2 University of Milan, Aldo Ravelli” Center For Nanotechnology And Neurostimulation, Milan, Italy; 3 Università degli Studi di Milano, Health Science Department, Milano, Italy; 4 Università degli studi di Trieste, Dipartimento Universitario Clinico Di Scienze Mediche Chirurgiche E Della Salute, Trieste, Italy; 5 University of Turin, San Luigi Gonzaga Hospital, Turin, Italy; 6 IRCCS Ospedale Policlinico San Martino, Genoa, Italy, Department Of Neuroscience, Rehabilitation, Ophthalmology, Genetics, Maternal And Child Health, Section Of Psychiatry, University Of Genoa, Genoa, Italy, Genoa, Italy; 7 IRCCS Orthopedic Institute Galeazzi, Department Of Functional Neurosurgery, Tourette Center, Milan, Italy; 8 University of Milan, “aldo Ravelli” Center For Nanotechnology And Neurostimulation, Milan, Italy; 9 Stanford University, Department Of Psychiatry And Behavioral Sciences, Stanford, United States of America; 10 University of Milan, “centro Per Lo Studio Dei Meccanismi Molecolari Alla Base Delle Patologie Neuro-psico-geriatriche”, Milan, Italy

**Keywords:** psychopharmacology, obsessive compulsive disorder, tic disorder, Treatment Resistance

## Abstract

**Introduction:**

Obsessive Compulsive Disorder (OCD) and Tic Disorder (TD) are two highly disabling, comorbid and difficult-to-treat conditions. DSM-5 acknowledged a new “tic-related” specifier for OCD, i.e., Obsessive-Compulsive Tic-related Disorder (OCTD), which may show poor treatment response.

**Objectives:**

The aim of the present study was to evaluate rates and clinical correlates of response, remission and resistance to treatment in a large multicentre sample of OCD patients with versus without tics.

**Methods:**

398 patients with a DSM-5 diagnosis of OCD with and without comorbid TD was assessed from ten psychiatric departments across Italy. Treatment response profiles in the whole sample were analysed comparing the rates of response, remission and treatment-resistance as well as related clinical features. Multivariate logistic regressions were performed to highlight possible treatment response related factors.

**Results:**

Later ages of onset of TD and OCD were found in the remission group. Moreover, significantly higher rates of psychiatric comorbidities, TD, and lifetime suicidal ideation and attempts were associated to the treatment-resistant group, with larger degrees of perceived worsened quality of life and family involvement.

**Conclusions:**

While remission was related to later ages of OCD and TD onset, specific clinical factors, such as early onset and presence of psychiatric comorbidities and concomitant TD, predicted a worse treatment response, with a significant impairment in quality of life for both patients and their caregivers. These findings suggest a worse profile of treatment response for patients with OCTD.

**Disclosure:**

No significant relationships.

